# Supplementary material for: Are carbon emissions trading and green financial instruments synergistic? -Comprehensive quantitative research based on content analysis
Source: PLoS One. 2024 Mar 7;19(3):e0298601. doi: 10.1371/journal.pone.0298601 (PMC10919601; doi:10.1371/journal.pone.0298601)
Supplement: S1 Data — (ZIP) [file pone.0298601.s001.zip › Data/地级市Z_F工作报告.zip!/┴╔─■/╕╖╨┬╩╨/└┤╫╘╣1⁄2╓┌║┼í╛┬φ┐╦╩2╛▌═°í┐_2009.docx]

各位代表：

　　现在，我代表市政府向大会报告工作，请予审议，并请市政协各位委员和其他列席同志提出意见。

　　一、2008年工作回顾

　　2008年，是不平凡的一年。在市委的正确领导下，市政府同全市人民一道，紧紧抓住“突破阜新”重大发展机遇，奋力拼搏，开拓创新，完成了市十四届人大一次会议确定的各项任务，在转型振兴的新阶段，谱写了阜新经济社会发展的新篇章。

　　——国民经济跃上新台阶，综合实力进一步增强。预计实现地区生产总值238亿元，增长15.2％，增幅高于全省平均水平，其中，一、二、三产业分别增长16.9％、15.2％和14.3％；地方财政一般预算收入14.6亿元，增长32.9％，增幅超过全省平均水平并创历史新高；国税、地税收入分别增长40.6%和30.6%，创历史最好水平；全社会固定资产投资140.5亿元，增长35%；直接利用外资5000万美元，超过前三年总和；城市居民人均可支配收入突破万元，农村居民人均纯收入超过5000元，分别增长16.1％和22.3％。经济快速发展的良好局面，进一步鼓舞了全市人民的信心，更加坚定了我们实现转型振兴宏伟目标的决心。

　　——项目建设迈出坚实步伐，“突破阜新”实现良好开局。一批牵动全局、事关长远的重大项目建设取得突破性进展。总投资247亿元的大唐煤制气项目获得国家前期工作批复，开工准备工作基本就绪。巴新铁路项目按计划实施，重点控制性工程进展顺利。总规划360万千瓦的风电项目建设步伐加快，新开工60万千瓦；华能一期10万千瓦风场并网发电运行良好；华能二期投资31亿元的30万千瓦风电项目当年开工，当年陆续并网发电，风能开发规模居全省前列。华电彰武发电公司成立运行。阜新双汇、阜新六和等农产品深加工企业规模不断扩大，银茂木业、振隆南瓜深加工等一批项目竣工投产。重点产业集群发展迅速。彰武林产品产业集群引进项目45个。清河门皮革产业集群建设全面启动。液压、铸造、氟化工产业集群建设取得明显成效。全年新开工投资千万元以上项目181个，其中亿元以上32个。在实施“突破阜新”战略中，“三大产业基地”、重点产业集群迸发出巨大的发展能量，为实现转型振兴奠定了坚实基础。

　　——工业经济持续快速发展，整体规模和水平大幅提升。预计规模以上工业增加值63亿元，继续保持20%以上的增长速度；实现税金14亿元，增长30%。骨干企业进一步发展壮大。重点培育的80户骨干企业销售收入增长26%，占规模以上工业的82%。全市销售收入超5000万元企业新增19户，达到61户；税金超千万元企业新增6户，达到15户。太克集团规模快速扩张，销售收入由上年的1.4亿元增加到10亿元；大金集团、华通管道公司等企业利润实现翻番。中省直企业对地方经济的拉动作用和税收贡献率进一步提高。阜矿集团总收入和地方纳税额分别增长33%和47%。阜新发电公司2台35万千瓦机组投入运行，发电量增长18.5%。阜新供电公司售电量增长10.9%，增幅居全省前列；电网建设投入7亿元，为历年之最。全年完成国有企业改制22户。民营经济增加值增长23%。

　　——招商引资成效显著，对外开放实现新跨越。省委、省政府将我市正式纳入沈阳经济区，为阜新经济社会发展搭建了更广阔的平台。创新招商理念，突出主题概念，精心组织赴香港、台湾、长三角、珠三角等地区招商引资。充分利用省直部门帮扶平台，一批项目成功签约。全年招商引资到位超过50亿元。韩国顺德防冻液、阜德利再生能源等一批外资项目落户阜新。外贸出口突破1亿美元，增长16%。对内对外交流与合作引向深入，与通辽市、辛集市结为友好城市，日本援助华东医院项目顺利实施。经济开发区、高新区直接利用外资分别达到1000万美元和832万美元，对外开放水平进一步提高。

　　——县区经济加快发展，新农村建设扎实推进。预计县区生产总值、地方财政一般预算收入和固定资产投资分别增长20％、40％和60％，增幅均超过全市平均水平。县区工业占全市规模以上工业比重达到52%。农业产业化龙头企业新增16户，达到80户。粮食产量突破40亿斤，获历史性大丰收。农业结构调整迈出新步伐。保护地生产实现规模化发展，总面积超过10万亩；花生种植129万亩，居全省之首；畜牧业规模化饲养水平走在全省前列。阜、彰两县被国家列为生猪调出补贴县。及时妥善处理“三聚氰胺”事件带来的冲击，保持全市奶业健康发展。循环农业发展取得新成效，新增“四位一体”生态模式户3350户，新建1座中型秸秆气化站。机械化保护性耕作100万亩。造林81万亩，集体林权制度主体改革基本完成。人工增雨能力得到加强。新农村建设“八大工程”全面推进，新增致富项目户4.1万户。落实强农惠农资金4.9亿元，比上年增长近1倍。

　　——服务业活力明显增强，城乡消费市场日益活跃。社会消费品零售总额突破100亿元，增长22.1%。阜新大商进入国内同行业百强，新天地广场开业运营，知名品牌兴隆大家庭落户时代广场。海州庙商业步行街主体工程完工。蔬菜综合批发市场、果品市场、水产品肉类批发市场等一批专业市场投入使用。“万村千乡”市场工程覆盖96%的行政村，“新网工程”新增农村连锁超市290家，农村现代流通网络初步形成。第二届“中国·阜新玛瑙节”及农副产品展销会、风味美食节等展会取得良好成效。旅游市场日益繁荣，旅游总收入增长52.7％。我市被确定为全国唯一的工业遗产旅游示范区。海州矿国家矿山公园主题广场完工。瑞应寺等景区功能日趋完善。敖包文化旅游资源开发取得新进展。金融支持经济发展的能力明显增强，全年新增贷款35亿元，增长17％，高于全省、全国平均水平。彰武金通村镇银行挂牌营业。2家小额贷款公司获省政府批准筹建。上市企业实现零的突破。

　　——城乡基础设施建设不断加强，城市功能进一步完善。沈阜、阜朝、阜铁高速公路建成通车，区位优势明显提升。“三位一体”规划建设取得新成效，北外环路、人民大街北段、北新路中段竣工通车，拓展了城市发展空间。高德公铁立交桥交付使用。西山路等11条街路改造完成，建设农村公路224公里，79%的行政村实现通油路，群众出行环境不断改善。改造市区供热管网29.4公里，新增供热能力500万平方米，供热保障水平进一步提高。九营子河治理工程形成水域面积7.3万平方米。引白水源一期主体工程达到通水条件，市区管网配套工程完成5.6公里。市污水处理厂正式运营。污水再利用工程全面启动。新增城市绿地163万平方米，绿化覆盖率达到42％。卫生城建设取得阶段性成果，市容市貌发生明显变化。节能减排扎实推进，关停2台10万千瓦低效机组，取缔市区6吨以下燃煤锅炉16座。万元地区生产总值能耗下降4.8%。城市环境空气质量明显提高。第一次污染源普查完成。我市被确定为全国唯一的资源型城市生态恢复试点市。

　　——重点民生工程有效实施，和谐阜新建设步伐加快。实现城镇实名制就业7万人，“零就业家庭”保持动态为零；城乡劳务输出26.3万人次。市创业指导中心成立，扶持下岗失业人员自主创业基地100个。棚户区改造二期工程105万平方米住宅楼竣工，2.9万户居民陆续迁入新居。采煤沉陷区治理二期工程14.8万平方米住宅楼主体完工。完成老旧住宅小区环境整治45万平方米。援助农村贫困户建房1435户。城镇职工基本养老保险和基本医疗保险参保率分别达到93%和87%，分别提高10个和7个百分点；新型农村合作医疗参合率达到100%。城镇居民基本医疗保险启动实施。9.6万名退休人员养老保险待遇和22万名城乡低保人员保障标准得到提高。积极处理改制企业遗留问题，维护了职工权益。清理偿还拖欠农民工工资2100万元。积极开展抗震救灾捐赠活动，我市向四川灾区捐款捐物达3000余万元。蓓蕾生命救助、健康快车等慈善活动深入开展，6500余名困难群众得到救助。新建区域性中心敬老院5所，新增养老福利机构8家，市儿童福利院工程竣工。重点实施82个贫困村整村推进扶贫，全市15万农村贫困人口稳定脱贫。解决7.4万农村人口安全饮水问题。建设农村卫生厕所3800座。社区“十分钟服务圈”试点取得明显成效。居民消费价格指数在4.8%以内。市政府向全市人民承诺的街路改造、农村贫困户建房等10件实事全部兑现。

　　——经济社会协调发展，社会事业全面进步。科技创新能力进一步增强。新增企业研发中心10个、省级高新技术企业4户，开发新产品46个，高新技术产业产值增长25%。辽工大与42户企业开展科技合作取得显著成效；煤炭自燃防治课题获得国家科技进步二等奖。新一轮“普九”取得积极进展，细河区率先通过 “双高普九”验收。海州高中通过“省级示范高中”评审。职业教育专业及实训基地建设进一步加强，毕业生就业率达到96%。民办教育稳步发展。全部免除城市义务教育阶段学生学杂费。新建、维修校舍15万平方米，建设九年寄宿制学校7所。人才智力引进工作扎实开展，“一村一名大学生”工程和“大学生服务辽西北计划”深入实施。机关事业单位人员录用全部实行公开招聘。“市长杯”职工技能大赛调动了广大职工提高技能的积极性。文化精品不断涌现，小说集《回望沙原》等作品荣获全国优秀儿童文学奖。“东蒙短调民歌”入选第二批国家级非物质文化遗产保护名录。新建乡镇综合文化站22个、农家书屋95个，送电影下乡6700场。20户以上自然村“有线电视村村通”工程全面完成，农村广播电视信号实现全覆盖。《走进玛瑙之都》专题片在央视播出。社区卫生服务机构覆盖率达到100％；新建甲级村卫生室100个；“万名医师支援农村卫生工程”覆盖一半乡镇。全民健身运动深入开展。市体育馆开工建设，市民健身中心、海州区篮球馆等投入使用。成功举办首届“中国·阜新篮球节”。阜新篮球学院和辽宁三沟篮球俱乐部成立。阜新健儿在北京奥运会上取得一金一铜的骄人战绩，为家乡争得了荣誉。

　　——民主法制建设进一步加强，政府自身建设取得新成绩。自觉接受人大依法监督和政协民主监督，办理人大代表建议214件、政协提案232件，办结率100%，满意率98％。以行政审批制度为重点的行政管理体制改革全面推进，取消调整行政审批事项444项。电子政务网络平台建设启动，政府信息查询中心建立，政府信息化建设和政务公开工作进入新的阶段。紧密围绕县区、项目和企业深化服务，依法行政、廉政建设和效能监察深入开展，机关作风和经济发展软环境进一步改善。实施科学化、精细化财政管理，优化财政支出结构，保证了民生等重点支出。审计监督不断加强。国有资产监管更加规范。国土资源管理工作有效保障了项目建设用地需求。　“平安阜新”建设深入推进。圆满完成奥运安保任务，依法严厉打击各种违法犯罪活动，社会治安综合治理成效明显，人民群众安全感日益增强。坚持领导接访和下访制度，一批民生问题得到妥善解决。通过市长接访和接听公开电话、市长信箱及《行风热线》等渠道，及时解决了市民反映的热点、难点问题。深入开展以煤矿、非煤矿山、危险化学品和消防、道路交通、食品药品等为重点的安全生产专项整治，安全生产形势总体保持平稳。应急管理体系进一步完善。第七届社区居委会换届选举工作顺利完成。启动实施第二次经济普查，完成第二次农业普查。落实党的民族宗教政策取得新成效。民族地区经济和社会发展不断加快。阜蒙县50周年县庆取得圆满成功。国防后备力量进一步加强，双拥共建工作扎实开展，军政军民关系更加紧密。

　　各位代表，一年来，我们遇到的困难超乎预料，付出的努力非同寻常，取得的成绩来之不易。这些成绩的取得，是市委正确领导的结果，是市人大依法监督和市政协民主监督的结果，是全市人民共同努力奋斗的结果。在此，我代表市政府，向全市各族人民，向各民主党派、工商联、无党派人士、各人民团体和社会各界人士，向中省直单位、驻阜部队、武警官兵和政法干警，向关心支持阜新发展的老领导、老同志，向所有为阜新发展作出贡献的海内外朋友们，表示衷心的感谢并致以崇高的敬意！

　　在看到成绩的同时，我们也清醒地认识到，全市经济社会发展中还存在一些矛盾和问题。特别是受国际金融危机影响，一些矛盾更加突出。主要是：缺少具有战略性、牵动性的大项目，扩大经济总量的任务十分艰巨；部分企业开工不足，就业压力加大；农业产业化整体水平不高，农民持续增收基础薄弱；财政刚性支出增多，收支矛盾依然突出；城乡基础设施不够完善，城市管理中一些突出问题需加快解决；个别行政部门改革创新意识不强，服务意识淡薄，发展环境有待进一步优化。我们诚恳地希望各位代表、各位委员对政府工作提出更多更好的批评意见和建议，帮助政府提高执政能力和服务水平，共同把阜新建设好、发展好。

　　二、2009年工作安排

　　2009年，是全面落实省委、省政府“突破辽西北”战略，推进转型振兴向更高目标迈进的关键之年。当前，国际金融危机日趋严峻，国内外市场形势发生了深刻变化。我市既面临着严峻的挑战，更面临着前所未有的发展机遇。中央经济工作会议确定了“保增长、扩内需、调结构”的工作方针，国家为力促经济平稳较快增长，采取了一系列坚强有力的措施。省委、省政府及时出台了扩大内需、增加投资的一系列积极政策，同时，作出了实施“突破辽西北”战略的重大决策。所有这些，都为我们战胜前进道路上的困难，保持超过全省平均水平的发展势头，推进转型振兴创造了有利条件。我们要坚定信心，鼓足干劲，团结奋斗，为实现我市在突破辽西北战略中“三年见成效，五年大变样”目标打下坚实基础。

　　按照市委十届七次全会的部署，今年政府工作的总体要求是：全面贯彻党的十七大和十七届三中全会精神，以邓小平理论和“三个代表”重要思想为指导，深入贯彻落实科学发展观，以“突破阜新”为主线，以保持经济平稳较快增长为首要任务，以解放思想、改革开放为动力，以推进重大项目建设为主攻方向，加快“三大产业基地”和重点产业集群建设步伐，进一步优化经济结构，转变经济发展方式，统筹城乡协调发展，着力改善民生，促进社会和谐，为早日实现转型振兴目标而努力奋斗。

　　今年全市经济社会发展主要预期目标是：地区生产总值285亿元，增长18％；全社会固定资产投资200亿元，增长42.3%；地方财政一般预算收入16.9亿元，增长15.3％；城市居民人均可支配收入1.1万元，增长10％；农村居民人均纯收入5600元，增长11％；城镇人口登记失业率控制在4.5%以内；万元地区生产总值能耗下降4.8%。

　　（一）紧紧抓住经济转型试点市和“突破辽西北”机遇，全力推进重大项目建设，加快工业经济发展

　　以重大项目建设为突破口，加快建设“三大产业基地”和重点产业集群，切实增强骨干企业发展能力，壮大工业经济总体实力。

　　加快重大项目建设步伐。全力推进大唐煤化工项目，年产40亿立方米煤制气项目开工建设。巴新铁路项目完成工程总量的50%。风电项目新开工60万千瓦，总装机容量达到72.5万千瓦。积极推进华电彰武电厂开工建设。阜新发电公司四期扩建项目力争核准。力达铸造、太恒铜业等20个投资亿元以上续建项目建成投产。明阳风电装备制造、森工木业等40个投资亿元以上项目开工建设。

　　突出抓好产业集群建设。以建设 “全国重要的板材家具制造基地” 和“中国北方皮革城”为目标，加大项目引进力度，彰武林产品产业集群、清河门皮革产业集群分别引进项目50个。液压、铸造、氟化工产业集群实现总量扩张。经济开发区、高新区加大招商引资力度，提高园区产业集聚度和投资密度。

　　保障工业经济健康平稳运行。积极应对国际金融危机的冲击，加强工业经济运行调度监控，强化对企业的政策扶持和金融支持，帮助企业渡过难关。引导企业加强管理，苦练内功，降低生产成本，培育自主品牌，调整产品结构，积极开拓市场，提升企业素质和市场竞争力。加大对重点骨干企业支持力度，促进企业做大做强。全力支持阜矿集团稳步发展煤炭产业，加快发展非煤产业，大力推进膨润土开发、玄武岩深加工和粉煤灰及煤层气综合利用等重点项目建设，尽快实现百亿元企业目标。

　　大力发展民营经济。进一步完善中小企业服务体系，加强中小企业融资服务中心建设，支持民营企业发展壮大。积极引导民营企业加快建立现代企业制度，培养高素质企业家队伍，提升民营企业档次和水平。制定政策措施，鼓励本地民间资本不断扩大在阜投资。民营经济增加值、实缴税金均增长20%以上。

　　（二）牢牢把握国家扩大内需机遇，加大固定资产投入，加快推进城乡基础设施建设

　　大力推进铁路建设。经过积极争取，时速350公里的京沈客运专线已被国家确定途经阜新，我们要积极配合建设好这条备受全市人民瞩目的高速铁路。开工建设沈阳经阜新至锦州铁路复线电气化工程，启动沈阳至彰武和阜新至沟帮子铁路前期工作。规划建设新邱至清河门城市轨道公共交通。完成彰武火车站改扩建工程。

　　加快高速公路建设。开工建设彰武至通辽、阜新至盘锦高速公路。积极推进阜新至奈曼高速公路前期工作。启动高孙路等3条高速公路出口路建设工程。进一步改善我市城际交通状况。

　　完善城乡道路交通网络。完成国省干道大中修173公里。开工建设南环路、西环路，城市外环框架基本形成。延伸煤城路、北外环路、迎宾大街、人民大街、中华路东段等主要干道，建设阜邱路、东新路，维修改造东风路等10条街路。五龙公铁立交桥建成通车。建设农村公路531公里，实现行政村全部通油路。改扩建市长途客运站，规划建设市区北、阜蒙县长途客运站，彰武、新邱长途客运站交付使用。

　　加大城市管网改造建设力度。城市二期供水管网和“三环制”供暖改造工程全面完工。改造建设城市排水管网和雨污分流系统。引白水源一期工程投入使用，二期工程前期工作加快推进。

　　加强城市水系建设。完善并实施城市水系规划。建设4座污水处理厂，筹建2处人工湿地污水处理系统，污水再利用工程投入使用。继续推进细河综合治理工程，九营子河城市段治理和蓄水工程完工，旱河水系形成水域5万平方米。

　　改造建设城乡电网。建设彰东、彰北风电场接入系统和西山、阜北、他本输变电工程，完成水泉变电站扩建工程。

　　加强公共服务基础设施建设。支持辽工大建设全国一流的职业技术学院。市体育馆、市工人文化宫二期工程完工。开工建设市图书馆、博物馆、会展中心、中国医大彰武附属医院，改扩建市中心医院、阜矿总医院。迁移新建市殡仪馆。

　　（三）以加入沈阳经济区为契机，以招商引资为重点，构筑对外开放新格局

　　充分利用沈阳经济区平台，加强区域经济合作，努力实现资源共享、优势互补、借力发展。加快“沈彰新区”建设，面向沈阳招商取得明显成效。启动沈阜经济产业带建设，积极承接装备制造、农产品加工等产业辐射和带动。全面加强与沈阳合作，在生态建设、产业配套、旅游开发和人才交流等方面合作取得积极进展。

　　进一步创新招商方式，突出产业集群和主题概念招商，充分发挥以商招商作用，提高招商引资效率和质量。精心组织好各项招商活动，引进一批国内外知名企业和上市公司。积极搭建招商平台，办好第四届“中国·阜新玛瑙博览会”和全国首届“花生博览会”。加强对重大项目、重点客商跟踪推进，提高合同履约率和项目开工率。全年完成招商引资60亿元，其中直接利用外资6000万美元，增长20%；新引进投资亿元以上项目30个、投资500万美元以上外资项目10个。

　　做好项目的研发储备，重点论证包装一批市场前景好、带动能力强、产业层次高的项目。强化考核评价，完善政策措施，调动各方面招商引资的积极性。加强以服务企业为中心的软环境建设，降低商务成本，提高服务水平。

　　优化出口产品结构，支持出口企业加强市场开发和出口基地建设，不断增强开拓国际市场的能力。全年出口创汇1.2亿美元。加强对外劳务输出，促进对外经济合作。

　　（四）统筹城乡协调发展，推进县区经济快速崛起，加快社会主义新农村建设

　　大力发展县区工业，壮大县区经济实力。支持县区加快培育主导产业和支柱产业，着力推进每个县区培育一个产值超50亿元的产业集群，引进建设一批重大项目，发展壮大一批骨干企业。提升农业产业化水平，重点引进培育一批成长性好、带动力强的龙头企业，全年新增规模以上龙头企业15户。县区生产总值增长35%以上，地方财政一般预算收入增长37%以上，生产经营型项目固定资产投资增长65％以上。

　　以“五化三有”为目标，加快现代农业基地建设。大力发展保护地生产，重点抓好设施农业小区建设，新建保护地3万亩以上。进一步调整种植业结构，着力发展特色种植业，扩大花生、南瓜、食用菌、树莓等种植面积。加快发展畜牧业，新建标准化畜禽小区150个，进一步壮大生猪等优势畜禽产业，规划建设全国重要的优质肉牛基地。加快大扁杏、速生丰产林等林果业基地建设。积极发展都市农业。加强农业标准化生产，保障农产品质量安全。继续推进“百万农民增收致富工程”，促进农民稳定增收。

　　加强农业基础设施建设，提高农业综合生产能力。推进一批农田水利工程建设，新增有效灌溉面积2万亩。加快推进农业机械化，完成保护性耕作115万亩。开工建设碱锅、巨龙湖水库除险加固工程。加强农村防灾减灾体系建设，进一步提高人工增雨能力。

　　推进农村改革，增强农村发展活力。统筹城乡规划、基础设施建设和公共服务，积极推进城乡一体化发展。继续开展城乡学校、医院对口支援帮扶和“百企带百村”等活动，搞好“城乡一体化”试点工作。鼓励引导农民发展专业合作社、专业经济技术协会等生产经营服务组织，大力发展专业大户，培育家庭农场等规模经营主体。积极开展土地经营权流转试点，建立县级土地经营权流转中心。深化集体林权制度配套改革，启动国有林场改革试点。

　　（五）以房地产业、物流业为重点，大力发展现代服务业，打造经济发展新的增长点

　　加快发展现代服务业和商贸流通业，全面提升第三产业发展水平。第三产业增加值106亿元，增长18%。社会消费品零售总额127亿元，增长18.5%。

　　大力发展房地产业。坚持统筹规划、优化布局、有序发展，全面推进住宅、工业、商业地产开发，更好地发挥房地产业对经济发展的拉动作用。突出地区特色，设计主题概念，积极引进知名房地产企业，开发适应不同层次需求的房地产项目。推动县域房地产业快速发展。全市房地产新开工面积180万平方米。

　　积极发展物流业。以建设辽西蒙东物流中心为目标，加快建设能源、交通、农副产品等物流园项目，引进知名物流企业，打造现代物流产业集聚区。

　　加快发展旅游业。推进工业遗产旅游项目开发，海州矿国家矿山公园正式开园。做好瑞应寺晋升4A级景区工作。整体开发高山子风电主题景区。整合资源，创新景区管理体制，促进旅游业快速发展。全年旅游总收入增长40％以上。

　　进一步发挥金融对促进经济发展的积极作用。挖掘现有金融机构发展潜力，不断扩大信贷投放规模。推进农村金融产品创新，引导各类金融机构支持农业农村发展。组建5家小额贷款公司和阜蒙县村镇银行。开办辽西票据再贴现中心。积极推进重点企业上市。利用信托融资、发行企业债券等方式，提高直接融资能力。做大做强市商业银行，重组6家城市信用社，实现跨地区经营。促进保险业创新服务方式，实现健康发展。

　　打造特色商业街区。时代广场、海州庙商业步行街和新邱区商业步行街投入运营。加快东市商业区改造，启动站前、迎宾大街等商业街区开发。积极引进五星级酒店项目和台湾大润发等大型超市项目，提升业态水平。培育发展特色餐饮业。举办各类独具特色的展会，促进会展业发展。加快商贸流通企业改制，激活传统商贸流通业。

　　加快各类市场建设。开工建设投资5亿元的汽配城和粮油批发市场等一批专业市场。统筹规划建设玛瑙城项目，发展壮大玛瑙等各类特色市场。改扩建哈尔套市场，重塑“哈尔套大集”品牌。“万村千乡”市场工程覆盖所有行政村。“新网工程”新增农村连锁超市100家。

　　促进通讯业健康快速发展。整合通讯资源，挖掘市场潜力，拓展业务范围，更好地满足社会需求。

　　千方百计增收节支。树立勤俭节约风尚，在全社会开展增收节支活动。强化对重点领域的税收征管，努力实现应收尽收。积极向上争取支持，保障重点支出。厉行节约，控制支出，提高财政资金使用水平。完善国有资产监管体系，促进国有资产保值增值。

　　（六）完善城市规划管理，加强资源节约和环境保护，努力建设生态文明

　　加强城市规划、建设和管理，努力打造宜居、宜业、宜商、宜游城市。高起点、高水平做好《城市总体规划》和《土地利用总体规划》修编工作，加快实施“三位一体”规划。建立健全科学高效的管理机制，提升城市管理水平。强化对重点区域的环境综合整治，下大力气改善市容市貌。完善政策措施，切实加强物业管理。力争3年内完成市中心区1万平方米以上老旧住宅小区环境整治。深入开展爱国卫生运动，争创国家卫生城。全年新增绿地150万平方米。消灭市区无灯路，建设一批公厕，着力解决群众关心的热点、难点问题。发展公共交通，加强出租车行业管理，保障城市交通顺畅。探索有效方式，加快经营城市步伐。积极推进城市公用企业建立现代企业制度。

　　加强资源节约和综合利用，大力发展循环经济。加大矿产资源勘探、开发和管理力度，推进资源深加工，促进资源高效利用。强化水资源保护和优化配置，建设节水型城市。依法管理土地资源，节约集约利用土地。整合热源，提高集中供热能力。规范管理粉煤灰综合利用，加快申博粉煤灰综合利用、金沙粉煤灰砌块等一批循环经济项目建设。科学开发利用矿井水。加快煤层气在工业领域的推广应用。抓好“四位一体”生态模式户、秸秆气化等循环农业项目建设。积极推进市垃圾处理厂二期工程和垃圾发电项目。

　　加大环境和生态保护力度，全面启动生态市和资源型城市生态恢复试点市建设。落实节能减排责任制，推行清洁、低碳生产。加强污染物总量控制，着力解决矿业生产污染环境、破坏植被等问题。加快推进海州矿地质灾害治理工程。集中开展城区烟尘专项整治，进一步改善大气环境质量。二氧化硫、化学需氧量排放分别下降3％和2.6％。完成辽西北边界防护林建设，启动实施高速公路及市区出口路两翼绿化，加大村屯绿化力度，全年植树造林70万亩。继续抓好封山禁牧工作。加强河道生态治理和沙化土地治理，有效控制水土流失。

　　（七）着力改善民生，促进社会和谐，让全市人民得到更多实惠

　　全方位、多渠道促进就业。实施积极的就业政策，大力支持劳动密集型企业发展，努力增加就业岗位。强化就业培训，促进就业观念转变和就业技能提高。以创业带动就业，加强创业基地建设，鼓励大学毕业生带头创业，扶持创业带头人300名以上。加强公益岗管理，提高就业援助水平。在全社会营造关心就业、支持就业的良好氛围，积极创造条件促进就业。实现城镇实名制就业5万人，确保“零就业家庭”至少一人就业；完成城乡劳务输出18万人次。

　　实施城乡保障性安居工程。全面完成棚户区改造二期配套工程和采煤沉陷区治理二期工程。积极争取支持，推进廉租房建设和平房区改造。3年内完成农村贫困户危房改造，今年改造2000户以上。

　　完善社会保障制度。依法推进社会保险扩面，城镇职工基本养老保险和基本医疗保险分别达到38.8万人和43万人。努力扩大城镇居民基本医疗保险覆盖面。进一步解决困难企业退休人员参加医疗保险问题。完善配套政策，健全城乡社会救助体系，适时提高城乡低保救助水平。推行社区民主评议机制，提高低保动态管理水平。加快养老服务社会化进程。大力发展慈善事业。

　　以整村推进扶贫开发为重点，实现10万农村贫困人口稳定脱贫。全面推进社区“十分钟服务圈”建设，让群众享受到更加便利的生活、文体、卫生和平安服务。启动实施“早餐便民工程”。加强改制企业履约监管，妥善解决改制遗留问题。加大农民工工资清欠力度，继续开展劳动关系和谐企业创建活动，保障劳动者合法权益。

　　（八）发展社会事业，加强民主法制建设，提高社会管理水平

　　加强精神文明建设。深入开展群众性精神文明创建活动，扎实推进“友爱阜新”建设。加强社会公德、职业道德、家庭美德、个人品德建设，提高人民群众道德素质和文明程度。

　　着力推进科技创新。扶持高新技术研发项目50个，加快推进林产品、液压产品等国家级研发中心建设，新增企业研发中心5个。加强与辽工大等高校的合作，加快科技成果转化。深入实施科技特派和进村入户工程，推广应用农业新技术。

　　努力办好人民满意的教育。继续推进新一轮“普九”，促进义务教育均衡发展。加快薄弱学校改造，在提高师资水平和改善办学条件等方面取得新成效。新建九年寄宿制学校2所，改造维修城乡校舍5万平方米。启动“操场建设工程”，改造维修25所中小学操场。深入开展城乡中小学“手拉手工程”，选派100名教师到农村支教。全面实施“教师素质提高工程”，做好中小学教师继续教育工作。以市场需求为导向，加强职业学校专业和实训基地建设，筹建彰武林业中等职业技术学校。促进民族教育发展。切实加强民办教育。支持辽工大人才引进、科技创新和项目研发，不断提高服务地方经济社会发展能力。

　　深入实施人才强市战略。坚持培养和引进并重，围绕企业发展和项目建设需求，培养适用人才；大力引进国内外人才智力，吸引高层次、创新型人才来阜创业。继续开展百名研究生引进工作，不断改善我市人才队伍结构。

　　加强医疗卫生服务体系建设。以重大传染病防控为重点，完善疾病预防控制体系和医疗救治体系。新型农村合作医疗资金使用率达到90%。建设200所甲级村卫生室。加大食品药品安全监管力度。切实做好人口和计划生育工作。继续开展“减盐”行动，倡导健康生活方式，不断提高全市人民健康水平。

　　促进文化体育事业繁荣发展。启动海州露天矿申报国家级工业遗产工作。现代京剧《血胆玛瑙》争创国家舞台精品剧目。推进文化体制改革。不断完善文化基础设施。新建乡镇综合文化站22个、农家书屋212个。推进文化信息资源共享工程进村入户。建设数字电视服务平台。广泛开展全民健身运动，办好市第七届运动会。深入开展“篮球月”等系列活动，建设好阜新篮球学院，加快篮球城建设步伐。

　　加强民主法制建设。自觉接受人大的依法监督和政协的民主监督，认真办理人大代表议案、建议和政协提案。加强与各民主党派、工商联、无党派人士的联系。支持工会、共青团、妇联等组织开展工作。支持法院、检察院依法履行职责。积极推进村务公开民主管理和农村社区建设试点工作。进一步巩固军政军民团结。

　　加大对民族自治地区的支持力度，促进民族事业加快发展。发挥宗教在促进社会和谐发展中的积极作用。

　　继续巩固“平安阜新”建设成果。全面落实社会治安综合治理各项措施，严厉打击各种违法犯罪活动，切实维护社会稳定。做好信访工作，积极预防和化解社会矛盾。深入开展“五五”普法。落实企业安全生产主体责任，提高企业安全管理水平。强化政府安全监管，加强对重点行业、重点领域安全隐患排查整治，坚决遏制重特大事故发生。

　　（九）适应时代发展要求，加强政府自身建设，提高执政能力

　　“突破阜新” 的新形势、新任务，对政府工作提出了新的更高的要求。我们要适应时代发展，推进改革创新，全面开创政府工作的新局面，切实承担起“突破阜新”的历史重任。

　　建设科学执政的法治政府。进一步建立和完善科学民主决策机制，努力使政府决策顺民心、合民意，让政府各项工作经得起人民的检验和历史的检验。不断创新政府管理方式，强化社会管理和公共服务职能。充分发挥市场配置资源的基础性作用，促进生产要素的有序高效流动。深化政务公开，增强政府工作透明度和公信力。坚持依法行政，严格按照法定权限和程序行使权力、履行职责，强化行政执法监督。加强社会信用体系建设，营造全社会诚信环境。

　　建设真抓实干的效能政府。以深入学习实践科学发展观活动为契机，促进新一轮思想大解放，增强改革创新能力，推进各项工作实现新突破。大力弘扬求真务实、真抓实干的作风，集中精力抓重点、抓大事。加强重大项目和重点工作的督促检查，抓好跟踪落实。强化部门之间协调配合，确保令行禁止、政令畅通，提高政府执行力。转变文风会风，提高会议质量和效率。加快电子政务建设，提高信息化水平。完善绩效考核内容和方法，科学确定考核指标体系，注重考核结果的应用，切实增强导向作用。

　　建设心系百姓的责任政府。始终把改善民生作为政府工作的出发点和落脚点，时刻把百姓安危冷暖放在心上，满怀深厚的感情，深入到贫困群体中去，深入到困难多的地方去，倾听群众呼声，关心群众疾苦，解决群众困难。强化责任意识，用心做好每一项工作，使政府工作更多地惠及广大人民群众。进一步畅通诉求渠道，充分发挥市长公开电话、市长信箱等渠道作用，使群众的合理诉求得到及时妥善解决。建立健全有效应对供水、供热、供电等公共突发事件的工作机制，提高应急管理能力和水平。

　　建设人民满意的服务型政府。以服务企业、服务百姓、服务社会为重点，全面提高政府服务水平。深化行政管理体制改革，进一步简政放权，提高行政审批效率。强化服务意识，改进服务方式，坚持深入基层，提倡现场办公，解决实际问题。加强行政服务中心建设，完善工作机制，优化工作流程，努力打造优质高效、便捷规范的服务平台。建立完善公务员考核、奖惩、培训制度，努力提高公务员素质。

　　建设拒腐防变的廉洁政府。全面落实党风廉政建设责任制，扎实推进惩治和预防腐败体系建设，从源头上预防和惩治腐败。加强重点领域、重点工程、专项资金的财政监督、审计监督和廉政监察，严惩商业贿赂行为。以解决群众反映的突出问题为重点，坚决纠正行业不正之风，严肃查处各种违法违纪行为。各级领导干部要严格遵守廉洁自律各项规定，带头树立过紧日子的思想，继续保持艰苦奋斗的优良传统，切实做到清正廉洁，执政为民。

　　各位代表，“突破阜新”任务艰巨，使命光荣，责任重大。让我们在市委的正确领导下，紧紧团结和依靠全市各族人民，以更加旺盛的斗志，更加饱满的热情，更加务实的作风，顽强拼搏，锐意进取，为实现阜新转型振兴，为全市人民更加幸福而不懈奋斗！努力开创阜新经济社会发展的崭新局面，以优异成绩迎接建国60周年！
